# Twenty-year trends of potentially avoidable hospitalizations for hypertension in Switzerland

**DOI:** 10.1038/s41440-024-01853-x

**Published:** 2024-08-21

**Authors:** Pedro Marques-Vidal, Ko Ko Maung, Alexandre Gouveia

**Affiliations:** 1 Department of Medicine, Internal Medicine, Lausanne University Hospital (CHUV) and University of Lausanne, Lausanne, Switzerland; 2 Centre for Primary Care and Public Health (Unisanté), University of Lausanne, Lausanne, Switzerland

**Keywords:** Hypertension, Epidemiology, Potentially avoidable hospitalizations, Administrative data

## Abstract

We assessed the trends, characteristics, and consequences of potentially avoidable hospitalizations (PAH) for hypertension in Switzerland, for the period 1998 to 2018. Data from 117,507 hospitalizations (62.1% women), minimum age 20 years. Hospitalizations with hypertension as the main cause for admission were eligible. PAH for hypertension was defined according to the Organization for Economic Cooperation and Development criteria. The age-standardized rates of PAH for hypertension increased from 43 in 1998 to 81 per 100,000 in 2004, to decrease to 57 per 100,000 inhabitants in 2018. Compared to non-PAH, patients with PAH for hypertension were younger, more frequently women (66.9% vs. 56.7%), non-Swiss nationals (15.9% vs. 10.9%), were more frequently admitted as an emergency (78.9% vs. 59.5%), and by the patient’s initiative (33.1% vs. 14.1%). Patients with PAH had also fewer comorbidities, as per the Charlson’s index. Patients with PAH for hypertension were more frequently hospitalized in a semi-private or private setting, stayed less frequently in the intensive care unit (4.6% vs. 7.3%), were discharged more frequently home (91.4% vs. 73.0%), and had a shorter length of stay than patients with non-PAH for hypertension: median and [interquartile range] 5 [3–8] vs. 9 [4–15] days. In 2018, the total costs of PAH were estimated at 16.5 million CHF, corresponding to a median cost of 4936 [4445–4961] Swiss Francs per stay. We conclude that in Switzerland, PAH have increased, represent a considerable fraction of hospitalizations for hypertension, and carry a non-negligible health cost.

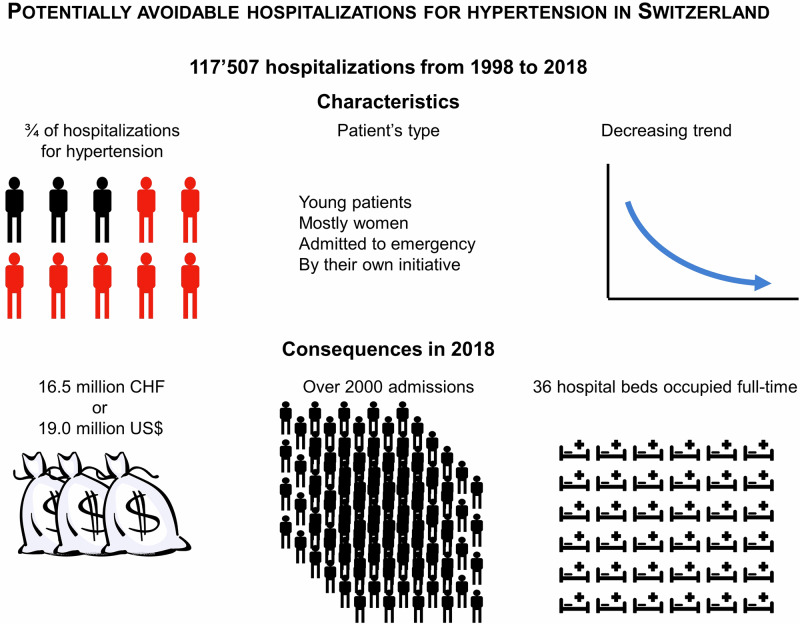

## Introduction

Hypertension is a major cause of cardiovascular disease, being responsible for over 10 million deaths and 200 million disability-adjusted life-years worldwide [1, 2]. Hypertension can be adequately management in the ambulatory setting, and international guidelines have been provided for adequate management of this condition [3]. Still, even in a country with a strong health system such as Switzerland, control rates of hypertension are far from optimal [4–6]. As most cases of hypertension can be managed in clinical practice, hospitalization for hypertension should be limited to patients with resistant or comorbidity-associated hypertension. Hypertension is considered as one of the ambulatory care-sensitive conditions (ACSCs) for which hospitalizations could be substantially prevented with the timely and effective primary care. Evidence indicates that comprehensive primary care is associated with a reduction in hospitalizations for ACSCs, which are considered as potentially avoidable hospitalizations (PAH) [7]. The Organization for Economic Cooperation and Development (OECD) defined PAH for hypertension as all non-maternal/non-neonatal hospital admissions with principal diagnosis code of hypertension, without in-hospital death, not resulting from a transfer from another acute care institution, not occurring during pregnancy, or not needing any cardiac procedure [8]. However, the trends in the number of PAH for hypertension vary globally [9–11]. As the management of hypertension is still perfectible in Switzerland, we searched whether PAH for hypertension is also high.

Hence, we aimed to assess the trends, characteristics, and consequences of PAH for hypertension in Switzerland, using data from 1998 to 2018.

## Methods

### Data

Hospital discharge data from 1998 to 2018 was provided by the Swiss Federal Office of Statistics (www.bfs.admin.ch) to conduct this specific study (contract 200291, reference 622.120-00023/00015). The data covers 98% of public and private hospitals within Switzerland and includes all stays for each hospital, even those that last less than 24 h [12]. The database contains information on the main cause for hospitalization, the comorbidities, specific surgical or medical procedures, vital status at discharge, and some socio-economic data, but no information regarding individual clinical data such as anthropometrics, blood pressure, or laboratory values, as well as no data regarding drug treatment. The main cause for hospitalization and the comorbidities were coded using the 10^th^ revision of the International Classification of Diseases (ICD-10) of the World Health Organization (WHO). The procedures were coded using the Swiss classification of surgical interventions (CHOP) [13]. Besides the ICD-10 and CHOP codes, the database also contains information regarding gender, age (categorized into 5-year groups), administrative regions (26 Swiss cantons), decision of admission (i.e., patient’s or doctor’s initiative, others), type of admission (planned or emergency), type of room (infirmary, semi-private or private), intensive care unit (ICU) stay and length of stay (LOS). For this study, the age categories were collapsed into 10-year groups, and the 26 cantons were attributed to the seven administrative regions, i.e., Leman, Mittelland, Northwest, Zurich, Eastern, Central, and Ticino), see supplementary Fig. 1.

The Charlson index was computed as indicated by Halfon et al. [14] and was used to assess the importance of comorbidities. The index was computed using data from the current hospitalization and patients were categorized into 0–1, 2–3 and 4+ score values.

### Potentially avoidable hospitalizations

The definition for a PAH for hypertension was obtained according to the international OECD Health Care Quality Indicators Project criteria [8] (Supplementary Table 1).

### Consequences of potentially avoidable hospitalizations

The total number of days due to PAH was computed for each year. This number was then divided by 365 to obtain the number of beds that would be theoretically solely dedicated to PAH during that year.

Costs were computed using the Swiss Diagnosis-Related Group (DRG) system. As the value of the DRG point varies according to canton and it was not possible to obtain the values for each year from each canton, costs in Swiss Francs (CHF, 1 CHF = 1.02 € or 1.10 US$ as of 8th May 2024) were computed by applying the cantons’ DRG value for year 2018 as performed previously [15].

### Inclusion and exclusion criteria

Only data related to adults (i.e., being at least in age group 20–24) and with an ICD-10 code of hypertension as the main cause of admission were eligible for analysis. Patients coming from outside of Switzerland or hospitalizations with missing covariates were excluded.

### Statistical analysis

Analysis was conducted using Stata version 18 for Windows® (Stata Corp, College Station, TX, USA). Descriptive results were expressed as number of hospitalizations (percentage) or as median [interquartile range]. Standardized rates of PAH for each calendar year were computed via direct standardization using the 2013 standard EU population. Between-group comparisons were performed using chi-square for categorical variables and Kruskal–Wallis test for continuous variables. Multivariable analyses were performed using logistic regression for categorical variables and results were expressed as odds ratio and (95% confidence interval). Multivariable analysis of LOS and costs was performed on log-transformed data using analysis of variance; values were back-transformed and expressed as mean ± standard error. Due to the sample size, statistical significance was considered for a two-sided test with *p* < 0.001.

### Ethics statement

The data of the Swiss Federal Office of Statistics is available for research purposes and therefore neither specific individual consent nor authorization from an Ethics Committee is needed.

### Patient and public involvement statement

Patients or the public were not involved in the design, or conduct, or reporting, or dissemination plans of our research.

## Results

### Potentially avoidable hospitalizations

**Fig. 1:**
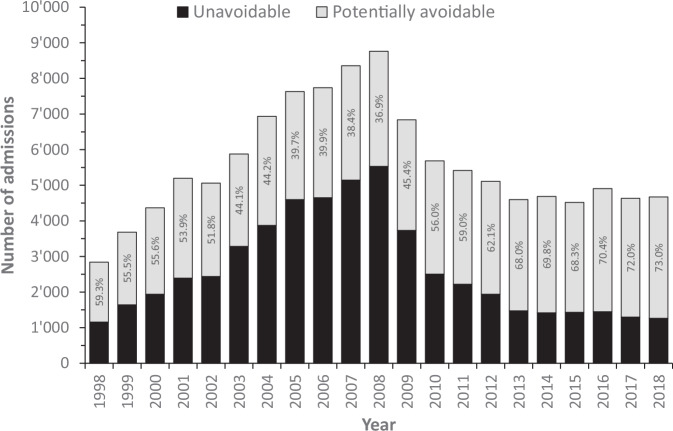
Yearly number of admissions for hypertension and percentage of potentially avoidable hospitalisations for hypertension in Switzerland, for period 1998 to 2018

**Fig. 2:**
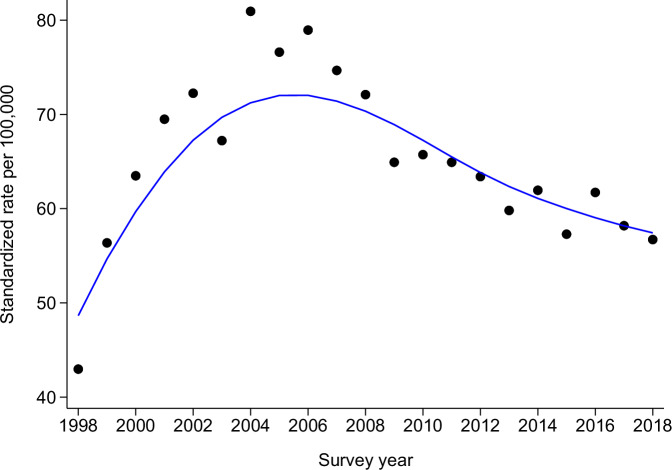
Trends in potentially avoidable hospitalisations for hypertension in Switzerland, for period 1998 to 2018, per 100,000 inhabitants, standardized using the European population

Of the initial 24,644,299 admissions for period 1998–2018, 130,502 (0.53%) had hypertension as the main cause of admission. The selection procedure is provided in Supplementary Fig. 2; the main cause for exclusion was missing data. The yearly distribution of the admissions for hypertension and the corresponding percentage of PAH are indicated in Fig. 1. The number of admissions for hypertension increased from 2843 in 1998 to 8761 in 2008, to decrease to 4671 in 2018. The percentage of admissions for hypertension considered as PAH was 59.3% in 1998, decreasing to 36.9% in 2008, to increase to 73.0% in 2018. Similar findings were obtained when the rate of PAH for hypertension was standardized on the European population, the rates increasing from 43 in 1998 to 81 per 100,000 in 2004, to decrease to 57 per 100,000 inhabitants in 2018 (Fig. 2).

The characteristics of the PAH for hypertension are summarized in Table 1. Patients with PAH for hypertension were younger, more frequently women, non-Swiss nationals, were more frequently admitted as an emergency, and by the patient’s initiative; patients with PAH had also less comorbidities, as indicated by the Charlson’s index categories. Regarding administrative regions, Eastern Switzerland, Central Switzerland, and Ticino had a higher frequency of PAH. Those differences were further confirmed after multivariable analysis (Table 1).

**Table 1:** Bivariate and multivariable analysis of the characteristics of potentially avoidable hospitalisations for hypertension, Switzerland, period 1998–2018

	Other hospitalizations for hypertension	Potentially avoidable hospitalizations	*P*-value	OR for potentially avoidable	*P*-value
*N*	55,372	62,135			
Women	31,377 (56.7)	41,565 (66.9)	<0.001	1.45 (1.41–1.49)	<0.001
Age group			<0.001		
[20–30]	150 (0.3)	388 (0.6)		1.74 (1.41–2.15)	<0.001
[30–40]	458 (0.8)	1244 (2.0)		1.84 (1.63–2.08)	<0.001
[40–50]	1339 (2.4)	3984 (6.4)		2.15 (1.99–2.31)	<0.001
[50–60]	3990 (7.2)	8013 (12.9)		1.65 (1.57–1.74)	<0.001
[60–70]	8060 (14.6)	11,639 (18.7)		1.31 (1.26–1.36)	<0.001
[70–80]	16,002 (28.9)	17,017 (27.4)		1 (reference)	
[80–90]	20,349 (36.8)	16,602 (26.7)		0.72 (0.69–0.74)	<0.001
[90+	5024 (9.1)	3248 (5.2)		0.50 (0.47–0.53)	<0.001
Non-Swiss national	6043 (10.9)	9901 (15.9)	<0.001	1.07 (1.03–1.12)	0.001
Administrative region			<0.001		
Leman	8018 (14.5)	6520 (10.5)		0.92 (0.88–0.96)	0.001
Mittelland	13,135 (23.7)	12,415 (20.0)		1 (reference)	
Northwest	9990 (18.0)	12,565 (20.2)		1.35 (1.30–1.41)	<0.001
Zurich	9860 (17.8)	10,013 (16.1)		1.11 (1.07–1.16)	<0.001
Eastern	7408 (13.4)	10,328 (16.6)		1.39 (1.33–1.46)	<0.001
Central	3624 (6.5)	5164 (8.3)		1.55 (1.47–1.65)	<0.001
Tessin	3337 (6.0)	5130 (8.3)		2.18 (2.06–2.31)	<0.001
No health insurance	1418 (2.6)	1734 (2.8)	0.015	1.08 (0.99–1.17)	0.070
Emergency admission	32,958 (59.5)	49,048 (78.9)	<0.001	2.83 (2.74–2.92)	<0.001
Decision of admission			<0.001		
Patient’s initiative	7827 (14.1)	20,550 (33.1)		2.20 (2.12–2.28)	<0.001
Ambulance or police	8596 (15.5)	10,123 (16.3)		1.30 (1.25–1.35)	<0.001
Doctor	37,692 (68.1)	31,049 (50.0)		1 (reference)	
Other	1257 (2.3)	413 (0.7)		0.42 (0.37–0.47)	<0.001
Charlson’s index categories			<0.001		
0–1	26,020 (47.0)	52,067 (83.8)		1 (reference)	
2–3	17,786 (32.1)	8121 (13.1)		0.22 (0.22–0.23)	<0.001
4+	11,566 (20.9)	1947 (3.1)		0.08 (0.07–0.08)	<0.001

All results are expressed as number of admissions and (%) for bivariate analysis and as odds ratio and (95% confidence interval) for multivariable analysis. Between-group comparisons performed using chi-square for bivariate analysis and logistic regression for multivariable analysis. For bivariate analysis, all comparisons are significant at *p* < 0.001. Logistic regression model was further adjusted for calendar year

*OR* odds ratio

### Consequences of potentially avoidable hospitalizations

The management and outcome of PAH is summarized in Table 2. Patients with PAH for hypertension were more frequently hospitalized in a semi-private setting, stayed less frequently in the ICU, were discharged more frequently home, and had a shorter LOS than patients with non-PAH for hypertension. The shorter LOS was confirmed after multivariable adjustment; 5.4 ± 1.0 vs. 4.5 ± 1.0 days for non-PAH and PAH, respectively, *p* < 0.001.

**Table 2:** Consequences of potentially avoidable hospitalizations for hypertension, Switzerland, 1998–2018

	Other hospitalizations for hypertension	Potentially avoidable hospitalizations	*P*-value
*N*	55,372	62,135	
Type of hospital stay (%)			<0.001
Infirmary	42,941 (77.6)	46,255 (74.4)	
Semi-private	8343 (15.1)	10,416 (16.8)	
Private	4088 (7.4)	5464 (8.8)	
Intensive care unit stay (%)	4038 (7.3)	2865 (4.6)	<0.001
Destination at discharge (%)			<0.001
Home	40,424 (73.0)	56,776 (91.4)	
Medical home	5405 (9.8)	2229 (3.6)	
Other	6349 (11.5)	3130 (5.0)	
Deceased	3194 (5.8)	0 (0)	
Length of stay (days)	9 [4–15]	5 [3–8]	<0.001

Results are expressed as number of admissions (percentage) for categorical variables and as median [interquartile range] for continuous variables. Between-group comparisons performed using chi-square for categorical variables and Kruskal–Wallis test for continuous variables

The trends in total number of hospital days due to PAH for hypertension are indicated in Supplementary Fig. 3. PAH for hypertension represented 17,931 hospital days in 1998, and this number increased to 27,831 in 2001, with a decreasing trend afterwards, to reach 13,380 days in 2018. This last figure represented between 36 and 37 beds fully occupied during the whole year 2018 for PAH for hypertension.

In the same year 2018, the costs of PAH were estimated at 16.5 million CHF, corresponding to a median cost of 4936 CHF per stay [interquartile range (IQR): 4445–4961 CHF], lower than for non-PAH: median and (IQR) 8271 (4752–9649) CHF. This difference persisted after adjusting for gender, age category, nationality, administrative region, type of admission, Charlson score categories and ICU stay: average±standard error 5278 ± 1 vs. 7497 ± 1 CHF for PAH and non-PAH, respectively, *p* < 0.001.

## Discussion

Our results indicate that, in Switzerland, a sizable fraction of hospital admissions for hypertension could be avoidable. Our results also indicate that PAH for hypertension have a significant cost and mobilise health resources which could be used for other diseases.

### Potentially avoidable hospitalizations: characteristics and comparison with other countries

The number of PAH for hypertension increased from 1998 to 2008 and decreased afterwards. The reason for such a decrease is currently unknown; a possible explanation would be a change in coding procedures, but no precise reason can be provided. This decrease is comparable to the one observed for asthma, but not for chronic obstructive pulmonary disease (COPD) [15]. The standardized rates varied between 43 and 81 per 100,000 inhabitants, more than for asthma but less than for COPD [15]. Interestingly, the non-standardized rates for Switzerland (maximum 71.6 per 100,000 inhabitants in 2004) were considerably smaller than reported for Germany in 2008 (205.66 and 102.65 per 100,000 inhabitants for men and women, respectively) [16], suggesting a better management of hypertension in Switzerland compared to Germany.

PAH for hypertension corresponded to almost three quarters of all hospitalizations for hypertension in Switzerland. This percentage is considerably higher than reported for all PAH in Switzerland (4.3%) [17], in a study conducted in a single Swiss university hospital (13.1%) [14] or in a study focusing on Swiss nursing home residents (42%) [18]. This value is also higher than reported for all conditions in Mexico (9.26%) [19], Portugal (10.9%) [20] or the US (20%) [21]. Overall, our results suggest that PAH for hypertension represent a significant fraction of all hospital admissions for hypertension in Switzerland, and that there is a potential for decrease.

Patients with PAH were younger, more frequently women, and with a lower number of comorbidities, a finding also reported for PAH for asthma [15]. A possible explanation would be higher anxiety levels as reported for women with white coat hypertension [22], which would maintain those levels high and motivate the persons to attend the emergency ward.

Over three quarters of PAH were admitted on an emergency status, one third of them by the patient’s initiative. Those values are similar to those reported for other conditions in Switzerland [17]. Still, their LOS was shorter, and ICU use less frequent, suggesting that the condition was of a less serious degree.

PAH levels differed significantly between regions, a finding also reported elsewhere [15–17, 23]. The most likely explanations are regional variations in health services such as number of physicians, hospital supply, or access to those services [16, 17, 24].

In 2018, PAH for hypertension represented over 10 admissions per day and between 36 and 37 beds fully occupied for a whole year. Those values are similar as for PAH for asthma, but lower than for COPD [15]. The cost of a PAH was significantly lower than for a non-PAH, likely due to the more benign condition, as patients with PAH had less comorbidities, needed ICU less frequently, and had a shorter LOS. Still, the total cost of PAH amounted to over 16 million CHF per year, a value that could be partly saved had a fraction of the PAH be prevented.

### Implications for policymakers

In the last years of the study period, over two thirds of hospitalizations for hypertension could be potentially avoidable. Those PAH represent a significant cost and mobilize health resources that could be directed to more severe diseases. As many PAH are motivated by the patients themselves and occur in an emergency setting, it would be important that educational measures targeting the whole population be provided so that patients are advised to call their general practitioner or to attend an ambulatory consultation before going to the hospital.

### Strengths and weaknesses

This study was conducted in a nationwide setting, using data covering over 95% of all Swiss hospitals. The data was collected using a standardized procedure throughout time, thus allowing assessing trends.

This study also has some limitations. Firstly, although it was conducted at a nationwide level, its results might not apply to other health systems, and it would be of interest that similar studies be conducted in other countries. Secondly, we used the OECD definition for PAH, and it has been shown that results vary according to the definition applied [25]. Still, the OECD definition would apply to many countries, thus allowing comparisons with other studies. Thirdly, no individual data regarding several potential confounders such as socioeconomic status or clinical information such as blood pressure values was available [26]. Still, some variables such as absence of compulsory health insurance and stay in a semi-private or private room might serve as proxies for categorizing the socioeconomic status of patients. Fourthly, the study period ended in 2019, and it would have been interesting to include more recent years. Still, given the coronavirus disease pandemic and the corresponding changes in the health system, it would have been difficult to interpret the trends during this period. Fifthly, due to legal constraints, it was not possible to associate geographical information with hospital characteristics; hence, whether some types of health care organizations had higher rates of PAH could not be ascertained. Finally, it was not possible to assess if several PAH were attributed to the same patient [21]. Still, we believe that this would not radically change the findings regarding the characteristics of patients with PAH for hypertension, and it would not impact the health and economic burden of PAH for hypertension.

## Conclusion

In Switzerland, PAH for hypertension increased from 1998 to 2004 and decreased afterwards. Patients with PAH are younger, with few comorbidities, and many are hospitalized by their own initiative. PAH for hypertension represented almost three quarters of all hospital admissions for hypertension, corresponding to 37 beds fully occupied per year, with an annual cost over 16 million CHF.

## Supplementary information

Supplementary information

Supplementary Figure 1

Supplementary Figure 2

Supplementary Figure 3

Supplementary figure legend

## Data Availability

The existing datasets used are not publicly available as per contract but can be requested to the Swiss Federal office of statistics.

## References

[1] G. B. D. Risk Factor Collaborators. Global, regional, and national comparative risk assessment of 84 behavioural, environmental and occupational, and metabolic risks or clusters of risks for 195 countries and territories, 1990-2017: a systematic analysis for the Global Burden of Disease Study 2017. Lancet. 2018;392:1923–94. 30496105 10.1016/S0140-6736(18)32225-6 PMC6227755

[2] G. B. D. Risk Factors Collaborators. Global, regional, and national comparative risk assessment of 84 behavioural, environmental and occupational, and metabolic risks or clusters of risks, 1990-2016: a systematic analysis for the Global Burden of Disease Study 2016. Lancet. 2017;390:1345–422. 28919119 10.1016/S0140-6736(17)32366-8 PMC5614451

[3] Williams B, Mancia G, Spiering W, Agabiti Rosei E, Azizi M, Burnier M, et al. 2018 ESC/ESH Guidelines for the management of arterial hypertension: The Task Force for the management of arterial hypertension of the European Society of Cardiology and the European Society of Hypertension: The Task Force for the management of arterial hypertension of the European Society of Cardiology and the European Society of Hypertension. J Hypertens. 2018;36:1953–2041. 30234752 10.1097/HJH.0000000000001940

[4] Danon-Hersch N, Marques-Vidal P, Bovet P, Chiolero A, Paccaud F, Pecoud A, et al. Prevalence, awareness, treatment and control of high blood pressure in a Swiss city general population: the CoLaus study. Eur J Cardiovasc Prev Rehabil. 2009;16:66–72. 19188810 10.1097/HJR.0b013e32831e9511

[5] Marques-Vidal P, Chekanova V, Vaucher J. Association between genetic risk of high SBP and hypertension control: the CoLaus|PsyColaus study. J Hypertens. 2022;40:1388–93. 35703291 10.1097/HJH.0000000000003158 PMC10004752

[6] N. C. D. Risk Factor Collaboration. Worldwide trends in hypertension prevalence and progress in treatment and control from 1990 to 2019: a pooled analysis of 1201 population-representative studies with 104 million participants. Lancet. 2021;398:957–80. 34450083 10.1016/S0140-6736(21)01330-1 PMC8446938

[7] Gao J, Moran E, Li YF, Almenoff PL. Predicting potentially avoidable hospitalizations. Med Care. 2014;52:164–71. 24374413 10.1097/MLR.0000000000000041

[8] Organization for Economic Cooperation and Development. Health Care Quality and Outcomes (HCQO) Indicators. 2022-23 Definitions. Paris, France: Organization for Economic Cooperation and Development; 2023.

[9] Agency for Healthcare Research and Quality. *Potentially Avoidable Hospitalizations* 2024. https://www.ahrq.gov/research/findings/nhqrdr/chartbooks/carecoordination/measure3.html. Accessed May 8^th^.

[10] Lu Y, Wang Y, Spatz ES, Onuma O, Nasir K, Rodriguez F, et al. National trends and disparities in hospitalization for acute hypertension among medicare beneficiaries (1999-2019). Circulation. 2021;144:1683–93. 34743531 10.1161/CIRCULATIONAHA.121.057056

[11] Chen S, Fu H, Jian W. Trends in avoidable hospitalizations in a developed City in eastern China: 2015 to 2018. BMC Health Serv Res. 2022;22:856. 35788227 10.1186/s12913-022-08275-w PMC9252061

[12] Federal Office of Statistics. Patients, hospitalisations [in French]. https://www.bfs.admin.ch/bfs/fr/home/statistiques/sante/systeme-sante/hopitaux/patients-hospitalisations.html. Accessed May 8th, 2024.

[13] Federal Office of Statistics. *Classification Suisse des Interventions Chirurgicales (CHOP)* 2022. https://www.bfs.admin.ch/bfs/fr/home/statistiques/sante/nomenclatures/medkk/instruments-codage-medical.assetdetail.5808564.html. Accessed 24 July 2023. Neuchâtel, Switzerland.

[14] Halfon P, Eggli Y, van Melle G, Chevalier J, Wasserfallen JB, Burnand B. Measuring potentially avoidable hospital readmissions. J Clin Epidemiol. 2002;55:573–87. 12063099 10.1016/s0895-4356(01)00521-2

[15] Gouveia A, Mauron C, Marques-Vidal P. Potentially avoidable hospitalizations by asthma and COPD in Switzerland from 1998 to 2018: a cross-sectional study. Healthcare (Basel). 2023;11:1229. 37174771 10.3390/healthcare11091229 PMC10178069

[16] Burgdorf F, Sundmacher L. Potentially avoidable hospital admissions in Germany: an analysis of factors influencing rates of ambulatory care sensitive hospitalizations. Dtsch Arztebl Int. 2014;111:215–23. 24739884 10.3238/arztebl.2014.0215 PMC3991158

[17] Gygli N, Zuniga F, Simon M. Regional variation of potentially avoidable hospitalisations in Switzerland: an observational study. BMC Health Serv Res. 2021;21:849. 34419031 10.1186/s12913-021-06876-5 PMC8380390

[18] Muench U, Simon M, Guerbaai RA, De Pietro C, Zeller A, Kressig RW, et al. Preventable hospitalizations from ambulatory care sensitive conditions in nursing homes: evidence from Switzerland. Int J Public Health. 2019;64:1273–81. 31482196 10.1007/s00038-019-01294-1 PMC6867979

[19] Valdes-Hernandez J, Reyes-Pablo AE, Canun-Serrano S, Navarrete-Hernandez E. Estudio de variabilidad geografica de las hospitalizaciones potencialmente evitables en Mexico durante tres quinquenios. Gac Med Mex. 2018;154:448–61. 30250313 10.24875/GMM.17003613

[20] Loureiro da Silva C, Rocha JV, Santana R. Economic and financial crisis based on Troika’s intervention and potentially avoidable hospitalizations: an ecological study in Portugal. BMC Health Serv Res. 2021;21:506. 34039326 10.1186/s12913-021-06475-4 PMC8152149

[21] McAndrew RM, Grabowski DC, Dangi A, Young GJ. Prevalence and patterns of potentially avoidable hospitalizations in the US long-term care setting. Int J Qual Health Care. 2016;28:104–9. 26705429 10.1093/intqhc/mzv110

[22] Johansson MAK, Ostgren CJ, Engvall J, Swahn E, Wijkman M, Nystrom FH. Relationships between cardiovascular risk factors and white-coat hypertension diagnosed by home blood pressure recordings in a middle-aged population. J Hypertens. 2021;39:2009–14. 33973957 10.1097/HJH.0000000000002888 PMC8452319

[23] Thygesen LC, Christiansen T, Garcia-Armesto S, Angulo-Pueyo E, Martinez-Lizaga N, Bernal-Delgado E, et al. Potentially avoidable hospitalizations in five European countries in 2009 and time trends from 2002 to 2009 based on administrative data. Eur J Public Health. 2015;25:35–43. 25690128 10.1093/eurpub/cku227

[24] Berlin C, Busato A, Rosemann T, Djalali S, Maessen M. Avoidable hospitalizations in Switzerland: a small area analysis on regional variation, density of physicians, hospital supply and rurality. BMC Health Serv Res. 2014;14:289. 24992827 10.1186/1472-6963-14-289 PMC4091658

[25] Bourret R, Mercier G, Mercier J, Jonquet O, De La Coussaye JE, Bousquet PJ, et al. Comparison of two methods to report potentially avoidable hospitalizations in France in 2012: a cross-sectional study. BMC Health Serv Res. 2015;15:4. 25608760 10.1186/s12913-014-0661-7 PMC4316643

[26] Wallar LE, De Prophetis E, Rosella LC. Socioeconomic inequalities in hospitalizations for chronic ambulatory care sensitive conditions: a systematic review of peer-reviewed literature, 1990-2018. Int J Equity Health. 2020;19:60. 32366253 10.1186/s12939-020-01160-0 PMC7197160

