# Supplementary material for: Twenty-year trends of potentially avoidable hospitalizations for hypertension in Switzerland
Source: Hypertens Res. 2024 Aug 21;47(10):2847–54. doi: 10.1038/s41440-024-01853-x (PMC11456504; doi:10.1038/s41440-024-01853-x)
Supplement: Supplementary file 1 — Supplementary information [file 41440_2024_1853_MOESM1_ESM.docx]

# Supplementary information

**Supplementary table 1**: criteria to define potentially avoidable hospitalizations for hypertension.

| **Hypertension** | **Potentially avoidable hospitalization** |
| --- | --- |
| And ICD-10 code beginning with I1 | ICD-10 codes   - I10: essential (primary) hypertension - I119: Hypertensive heart disease without (congestive) heart failure - I129: Hypertensive renal disease without renal failure - I139: Hypertensive heart and renal disease, unspecified   Exclude cases   - where the patient died in hospital during the admission. - resulting from a transfer from another acute care institution (transfers-in). - with cardiac procedure codes in any field [any Swiss classification of surgical interventions (CHOP) code beginning with 35, 36 or 37] - with pregnancy, childbirth, and puerperium codes in any field - that are same day/day only admissions |
