# Supplementary figures and images for: Twenty-year trends of potentially avoidable hospitalizations for hypertension in Switzerland
Source: Hypertens Res. 2024 Aug 21;47(10):2847–54. doi: 10.1038/s41440-024-01853-x (PMC11456504; doi:10.1038/s41440-024-01853-x)

## Supplementary figure 1: the seven administrative regions of Switzerland

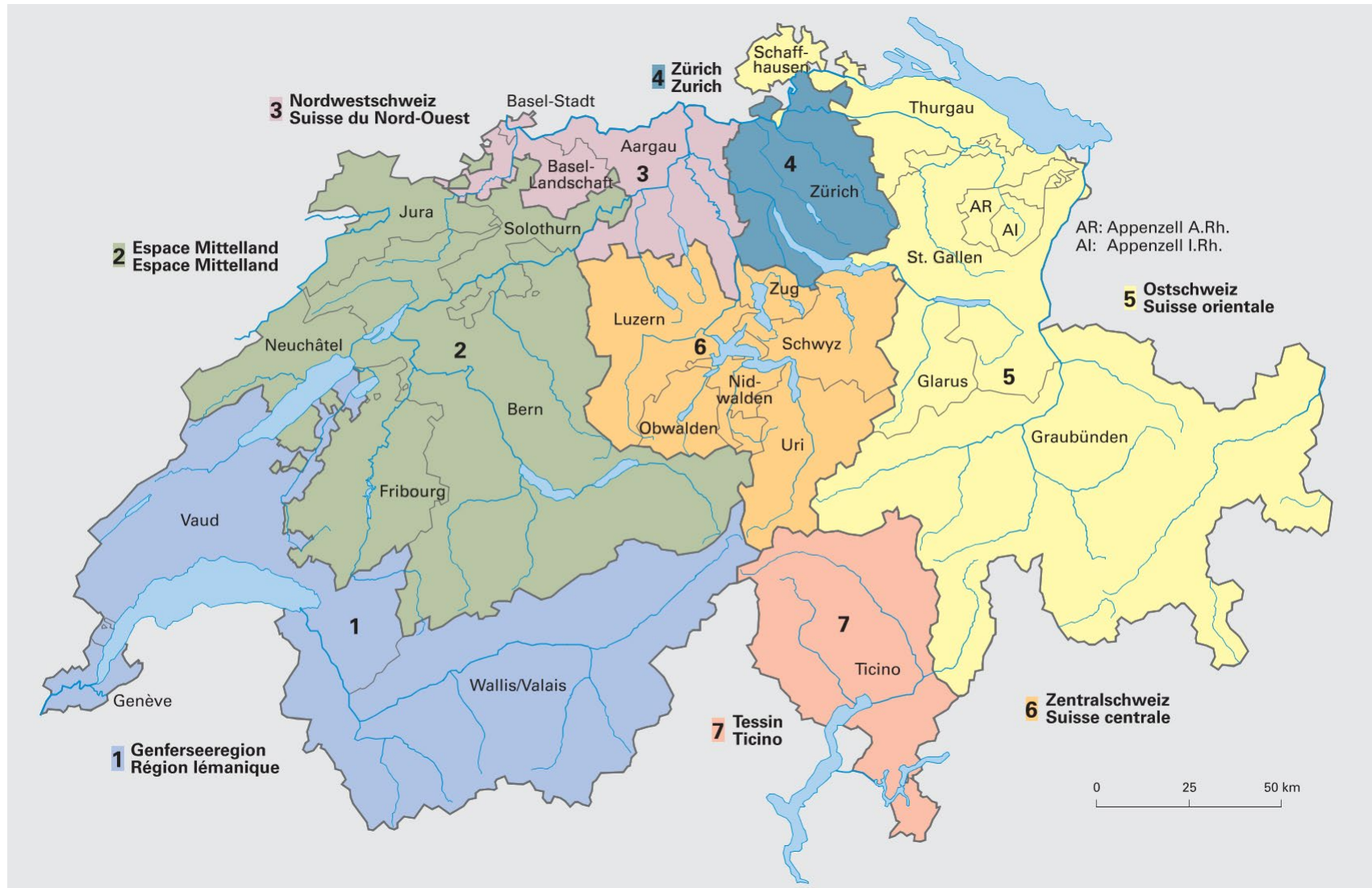

Supplement: Supplementary file 2 — Supplementary Figure 1 [file 41440_2024_1853_MOESM2_ESM.pdf]

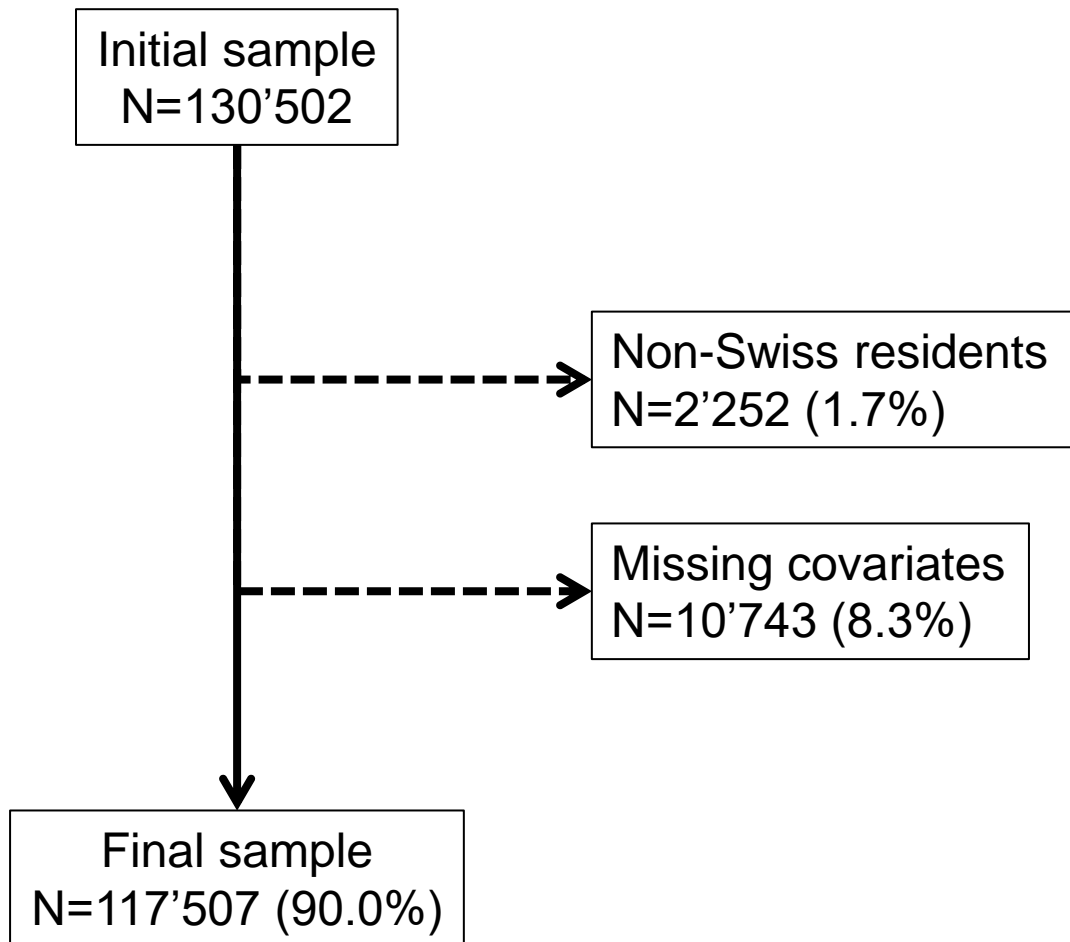

Supplement: Supplementary file 3 — Supplementary Figure 2 [file 41440_2024_1853_MOESM3_ESM.pdf]

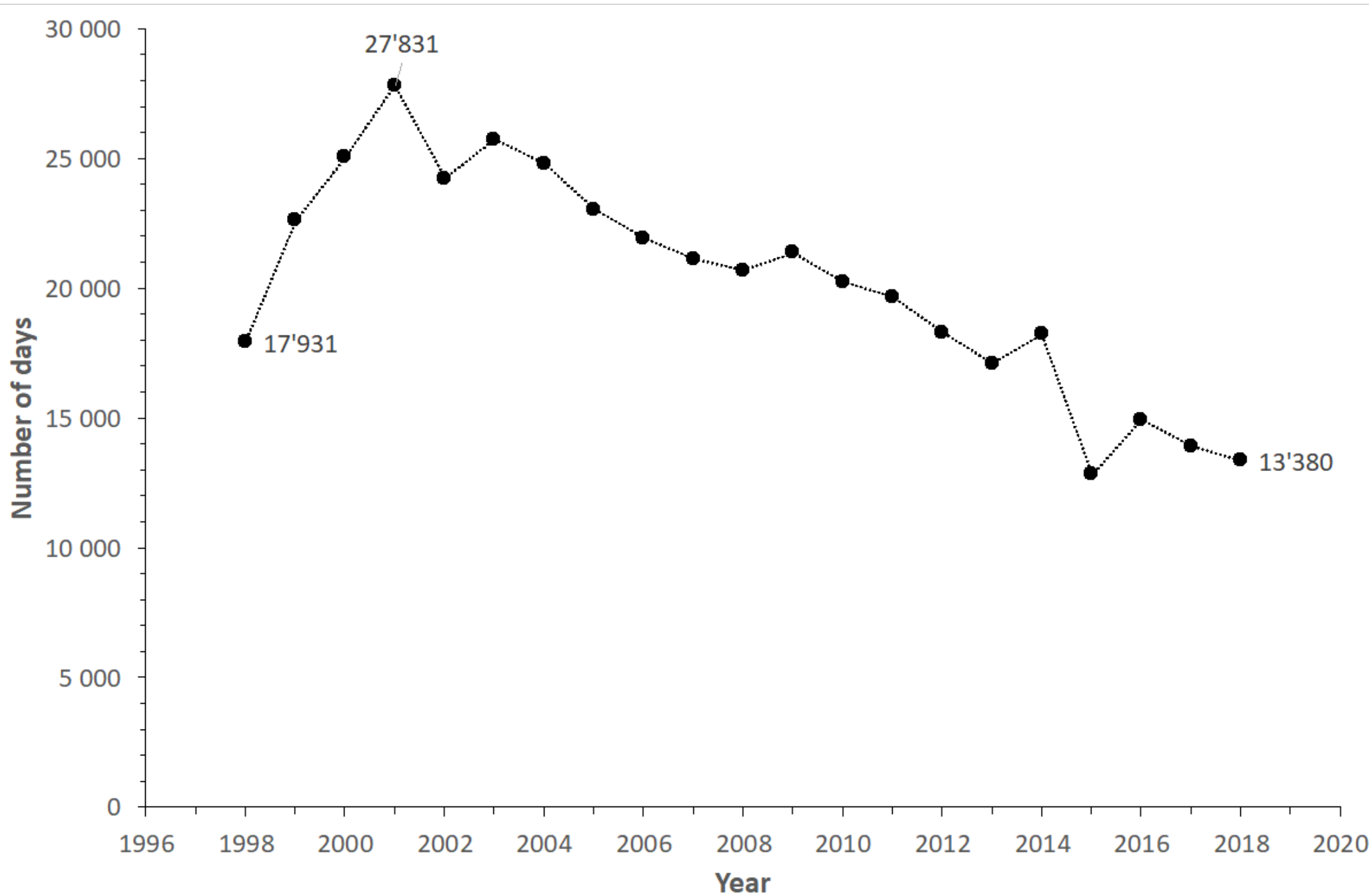

Supplement: Supplementary file 4 — Supplementary Figure 3 [file 41440_2024_1853_MOESM4_ESM.pdf]
