# Supplementary material for: Twenty-year trends of potentially avoidable hospitalizations for hypertension in Switzerland
Source: Hypertens Res. 2024 Aug 21;47(10):2847–54. doi: 10.1038/s41440-024-01853-x (PMC11456504; doi:10.1038/s41440-024-01853-x)
Supplement: Supplementary file 5 — Supplementary figure legend [file 41440_2024_1853_MOESM5_ESM.docx]

# Supplementary information

**Supplementary figure 1:** the seven administrative regions of Switzerland.

**Supplementary figure** **2**: selection procedure.

**Supplementary figure 3**: number of hospital days due to potentially avoidable hospitalisations for hypertension in Switzerland, for period 1998 to 2018.

**Supplementary table 1**: criteria to define potentially avoidable hospitalizations for hypertension.
